# Predicting ADHD in Children and Adolescents With Artificial Intelligence: A Scoping Review of Common Models

**DOI:** 10.1002/hsr2.71679

**Published:** 2025-12-21

**Authors:** Arefeh Ameri, Farzad Salmanizadeh, Hamidreza Samzadeh Kermani, Mohammad Mehdi Ghaemi

**Affiliations:** ^1^ Medical Informatics Research Center, Institute for Futures Studies in Health Kerman University of Medical Sciences Kerman Iran; ^2^ Faculty of Management and Medical Information Sciences Kerman University of Medical Sciences Kerman Iran

**Keywords:** artificial intelligence, attention‐deficit/hyperactivity disorder (ADHD), machine learning, random forest

## Abstract

**Background and Aims:**

Attention‐Deficit/Hyperactivity Disorder (ADHD) is one of the most prevalent neurodevelopmental disorders in childhood and adolescence, and its early diagnosis is essential for preventing long‐term cognitive and behavioural issues. Artificial intelligence (AI), as an emerging tool, holds significant potential for early prediction and detection of this disorder through analyzing clinical and behavioral data. This study reviews recent research on how AI is used to predict ADHD in children and adolescents.

**Methods:**

The PubMed, Scopus, Web of Science, and Embase databases were searched for articles on the use of artificial intelligence to predict ADHD through October 14, 2025. Data were collected using an extraction form based on the PRISMA‐ScR guidelines, and the findings were presented in figures and tables.

**Results:**

A total of 3981 records were identified through database searches, reduced to 1935 after duplicates were removed. Ultimately, 42 studies were included. The most frequently used AI methods were Random Forest (RF) (*n* = 22, 14.1%) and Support Vector Machine (SVM) (*n* = 20, 12.8%), all achieving an average prediction accuracy of over 80% for ADHD. Most studies relied on questionnaire‐based data (*n* = 28, 27.2%) and demographic information (*n* = 20; 19.4%), behavioral–cognitive characteristics of participants (*n* = 16, 15.5%), for ADHD prediction.

**Conclusion:**

AI, especially machine learning models like RF and LR, shows great potential for predicting and managing ADHD in children and teens. These methods could act as helpful tools for early diagnosis, leading to better cognitive, behavioral, and educational outcomes related to the disorder. However, the clinical translation of these AI‐based approaches requires attention to interpretability, workflow integration, and ethical considerations to ensure their safe and practical use in real‐world settings.

**Abbreviations**
ABCD: adolescent brain cognitive development
ADHD: attention‐deficit/hyperactivity disorder
AI: artificial intelligence
ANN: artificial neural network
ASD: autism spectrum disorder
AUC: area under the ROC curve
BiLSTM: bidirectional long short‐term memory
CNN: convolutional neural network
CV: cross‐validation
DNN: deep neural networks
DT: decision trees
EDI: early development instrument
EEG: electroencephalography
fMRI: functional magnetic resonance imaging
JBI: Joanna Briggs Institute
LASSO: least absolute shrinkage and selection operator
LOOCV: leave‐one‐out cross‐validation
LOSO‐CV: leave‐one‐subject‐out cross validation
LR: logistic regression
ML: machine learning
NSCH: national survey of children's health
ODD: oppositional defiant disorder
PRISMA‐ScR: preferred reporting items for systematic reviews and meta‐analyses extension for scoping reviews
RF: random forest
SVM: support vector machine
USA: United States of America

## 1 Introduction

Attention‐Deficit/Hyperactivity Disorder (ADHD) is one of the most common neurodevelopmental disorders in childhood, which can continue into adolescence and adulthood. The global prevalence of ADHD among children and adolescents has been reported to range from 5% to 12% [1].

Hyperactivity involves ongoing activity without fatigue, while inattention is marked by a lack of focus and distractibility [2]. Individuals with ADHD can display a wide range of symptoms, including difficulties with motivation, organization, and starting tasks; impaired concentration; irregular sleep–wake cycles; poor effort and persistence in completing tasks; forgetfulness; difficulty waiting their turn; interrupting others during conversations; missing details; and defying instructions [3]. In children and adolescents, ADHD can cause significant functional impairments such as poor academic performance, problematic interpersonal relationships, engagement in risky behaviors, and, in some cases, comorbid psychiatric disorders [4, 5]. Therefore, early prediction and diagnosis of ADHD can help develop appropriate therapeutic interventions, improve academic outcomes, and reduce behavioral difficulties in affected children.

Currently, early identification or prediction of ADHD risk mainly relies on traditional methods such as clinical interviews, parent/teacher questionnaires, and psychological tests [6, 7]. However, these methods are often time‐consuming, subjective, susceptible to human bias, and rely on the availability of experienced professionals, which can lead to delays in diagnosis, errors, and disparities in access to mental health care—especially in underserved areas or low‐income countries [6, 7]. To address these limitations, alternative diagnostic methods have been suggested, including functional magnetic resonance imaging (fMRI), which examines changes in cerebral blood flow and neural activity, and electroencephalography (EEG), which records real‐time electrical activity of the brain [8, 9]. However, these techniques face significant challenges, such as high costs, limited access to advanced equipment, the need for expert interpretation, and difficulty in detecting subtle variations or complex neural patterns related to ADHD. Additionally, results from these methods are often nonspecific and may overlap with other neuropsychiatric conditions, making it difficult to accurately predict ADHD risk at an early stage [10].

Recent advances in information technologies, especially artificial intelligence (AI), have introduced new approaches for predicting, diagnosing, treating, and managing various diseases. AI‐based methods, by analyzing large‐scale multimodal data (such as brain imaging, behavioral measures, genetic profiles, and environmental information), can identify hidden patterns and more accurate biomarkers [11]. Using advanced machine learning (ML) techniques, these systems enable personalized diagnosis, reduce human error, and offer more cost‐effective and time‐efficient solutions [11].

Previous studies have used AI in various contexts, including symptom identification [12], screening [13], classification [14], diagnosis [15, 16], and outcome prediction with both structured and unstructured data [15, 16, 17]. AI also has the potential to reduce inevitable human errors [16]. For example, AI can detect subtle data that might go unnoticed during behavioral observations, leading to more accurate data‐driven insights [18]. Given its capabilities in data processing, behavioral analysis, and outcome prediction, AI can be a powerful tool for predicting ADHD risk and identifying children at a higher likelihood of developing ADHD symptoms. ML algorithms and big data analytics enable AI‐based systems to identify behavioral patterns linked to ADHD and provide personalized strategies for managing the disorder. Several existing reviews have examined the role of AI in ADHD diagnosis and treatment [19, 20, 21, 22, 23]. However, early prediction and prognosis of ADHD remain particularly important, yet they have received little attention in previous AI‐driven reviews. Early prediction involves using AI techniques to estimate the likelihood of developing ADHD prior to a formal clinical diagnosis. This idea differs from diagnostic classification, which focuses on identifying ADHD in individuals who already exhibit clinically confirmed symptoms, and from treatment‐response prediction, which assesses outcomes among diagnosed patients [24]. Early prediction seeks to identify children and adolescents at increased risk based on behavioral, cognitive, neuroimaging, genetic, or environmental data collected during developmental stages [24]. This approach enables proactive monitoring and preventive interventions before significant functional impairments develop. Prompt identification of symptoms enables a faster and more accurate diagnostic process [25]. Therefore, early detection not only reduces long‐term complications but also enhances the quality of life for affected children [25]. The goal of our scoping review was to identify and summarize AI‐based methods and techniques that have been previously used to predict ADHD in children and adolescents. We excluded studies focused solely on diagnosis, classification, or treatment response, as our emphasis was on methods aimed at detecting elevated risk before a formal clinical diagnosis is made. Therefore, this review addressed the following question: which AI‐based techniques have been used to estimate the risk of ADHD before diagnosis in childhood and adolescence, and what are their methodological characteristics and predictive performance?

## 2 Methods

### 2.1 Study Design and Search Strategy

This scoping review was conducted and reported following the Preferred Reporting Items for Systematic reviews and meta‐analyses extension for Scoping Reviews (PRISMA‐ScR) guidelines [26]. A thorough literature search was performed in PubMed, Embase, Web of Science, and Scopus, with no time restrictions, up to December 16, 2024. To ensure literature currency, the search was re‐executed on October 14, 2025, using the original strategy and inclusion criteria, and any newly identified relevant studies were incorporated into this review. The search was carried out independently by a single researcher to identify potentially relevant publications. The search strategy included two sets of keywords: one related to AI and ML, and the other to ADHD (Appendix A). Synonyms within each set were combined using the OR operator, and the two sets of terms were combined using the AND operator.

### 2.2 Eligibility Criteria

The inclusion criteria for this study were as follows: (1) original research articles focusing on AI methods and their variants; (2) studies where these methods were used for the early prediction of ADHD in children and adolescents—that is, identifying individuals at risk of developing ADHD before or independently of a confirmed clinical diagnosis, using behavioral, cognitive, imaging, or multimodal data; (3) publications written in English.

Exclusion criteria included: descriptive studies, review articles, commentaries, editorials, and letters to the editor. Additionally, we excluded studies that focused solely on diagnostic classification of already‐diagnosed ADHD cases, treatment monitoring or outcome prediction in diagnosed patients, research conducted exclusively on adults, and conference papers without full‐text availability. In particular, we excluded neuroimaging‐based deep learning studies (e.g., Convolutional Neural Network (CNN), 3D‐CNN, autoencoders, and hybrid architectures applied to fMRI or MRI data) that classified previously diagnosed ADHD patients versus typically developing controls, as these studies do not estimate ADHD risk prior to clinical diagnosis and therefore fall outside the scope of early prediction.

### 2.3 Study Selection and Data Extraction

Following the database search, all retrieved records were imported into EndNote (version 21.0.1), and duplicate entries were identified and removed. In the first screening stage, potential relevant articles were independently assessed by two reviewers (A.A. and F.S.) based on their title and abstract, and any discrepancies were resolved through discussion and consensus. In the second stage, the full texts of articles that met all inclusion criteria were independently reviewed by the same two investigators (A.A. and F.S.), and data extraction was then performed. Data extraction was conducted using a structured form developed in Microsoft Excel. This form included items such as: authors' names, year of publication, study setting, study objectives, sample size and data set, applied AI method, performance evaluation and validation criteria, and main findings. Extracted data were cross‐checked by both reviewers (A.A. and F.S.), and any disagreements at any stage of the process were resolved through consultation with two additional researchers (M.Gh. and H.S.). The extracted data were analyzed using SPSS version 24 and presented as percentages, frequencies, means, standard deviations, ranges, and graphs. When reporting model performance metrics, we followed guidelines for transparent statistical reporting in the scoping reviews [26], providing ranges, means, and standard deviations where applicable to enhance reproducibility and interpretability.

### 2.4 Quality Assessment and Risk of Bias

The quality of the included studies was evaluated using standardized checklists developed by the Joanna Briggs Institute (JBI) [27]. The JBI offers specific critical appraisal tools tailored to different study designs, aimed at assessing potential bias sources across various research aspects, including sample size, study design, research methodology, confounding factors, and data analysis. In this review, the appropriate checklist was applied based on the type of each study. For each checklist item, responses were recorded as follows: 1 = Yes, 0 = No, U = Unclear, and N/A = Not applicable. Studies were then classified into three categories based on their percentage of the total score obtained:

• High risk of bias: score < 49%;

• Moderate risk of bias: score 50%–69%;

• Low risk of bias: score ≥ 70% [27].

Quality appraisal was performed independently by two reviewers (A.A. and F.S.). In cases of disagreement, consensus was reached after consultation with two additional reviewers (M.Gh. and H.S.).

## 3 Results

### 3.1 Study Characteristics

**Figure 1:**
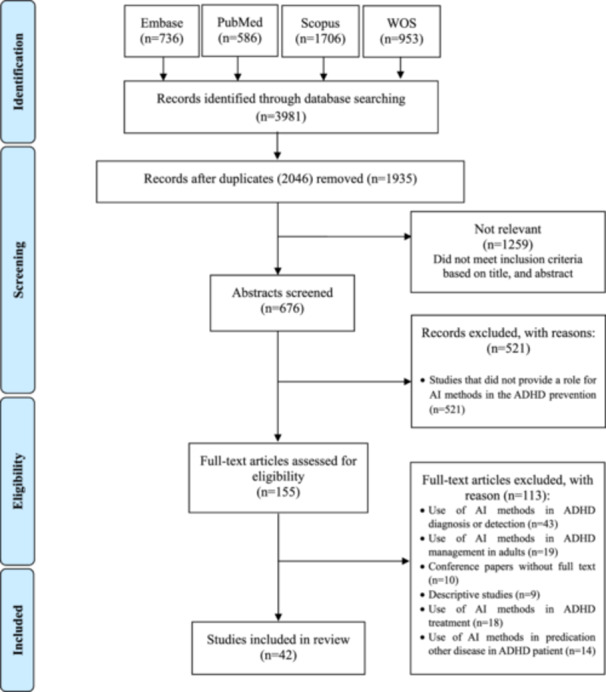
PRISMA‐ScR flow chart showing the search process. ADHD = attention deficit hyperactivity disorder, AI = artificial intelligence, PRISMA‐ScR = preferred reporting items for systematic reviews and meta‐analyses extension for scoping reviews.

The flowchart of the study selection process is shown in Figure 1. Initially, 3981 records were identified from four electronic databases. Of these, 2066 duplicates were removed. After screening titles and abstracts, 1259 articles were excluded as irrelevant, leaving 676 articles for further review. Among these, 155 articles were selected for full‐text assessment, and 113 were excluded based on predefined criteria. In the end, 42 studies met all eligibility requirements and were included in the review [28, 29, 30, 31, 32, 33, 34, 35, 36, 37, 38, 39, 40, 41, 42, 43, 44, 45, 46, 47, 48, 49, 50, 51, 52, 53, 54, 55, 56, 57, 58, 59, 60, 61, 62, 63, 64, 65, 66, 67, 68, 69] (Figure 1).

**Figure 2:**
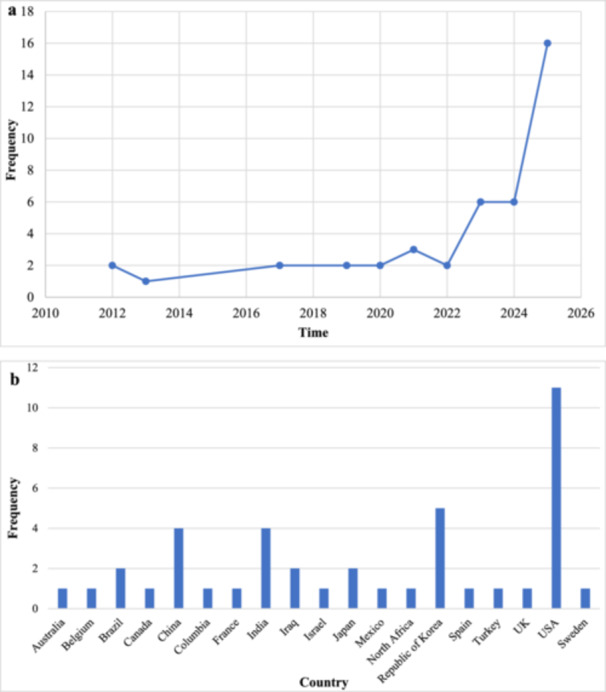
Study Characteristics. (a) Temporal distribution of the included studies, and (b) geographical distribution of the included studies. UK = United Kingdom, USA = United States of America.

Most of the included studies were published in 2025 (*n* = 16; 38.1%) [29, 30, 32, 34, 35, 40, 43, 44, 45, 47, 55, 56, 57, 59, 60, 69], 2024 (*n* = 6; 14.3%) [28, 49, 51, 52, 62, 67], and 2023 (*n* = 6; 14.3%) [33, 36, 38, 39, 46, 58] (Figure 2a). Regarding the country of origin, the majority of studies were conducted in the United States of America (USA) (*n* = 11; 26.2%) [30, 31, 36, 39, 41, 47, 49, 52, 54, 59, 64] (Figure 2b).

The number of participants in the included studies ranged from 24 [40] to 238,696 [38]. Additionally, 34 studies (81.0%) recruited only individuals with ADHD, while 8 studies (19.0%) included participants with ADHD alongside other neurological or psychiatric conditions, such as Oppositional Defiant Disorder (ODD), anxiety, autism spectrum disorder (ASD), and depression (Table 1). Further details of the included studies are provided in Appendix B.

**Table 1:** Characteristics of the included studies (*n* = 42).

Characteristics		Studies, *n* (%)	References
Number of participants	< 99	4 (9.5)	[37, 40, 41, 62]
	100–999	19 (45.2)	[28, 34, 36, 39, 42, 45, 47, 50, 51, 56, 57, 58, 60, 63, 64, 66, 69]
	> 1000	18 (42.9)	[29, 30, 31, 32, 33, 35, 44, 46, 48, 49, 52, 53, 54, 55, 59, 61, 65]
	Not reported	1 (2.4)	[43]
Participants' health conditions	Only ADHD	34 (81.0)	[28, 29, 30, 31, 34, 36, 39, 40, 42, 43, 44, 45, 46, 48, 49, 50, 51, 52, 53, 54, 56, 57, 59, 60, 61, 62, 63, 64, 65, 66, 69]
	ADHD and other diseases	8 (19.0)	[32, 33, 35, 41, 47, 55, 58]

### 3.2 Types of AI Models and Examined Features

**Figure 3:**
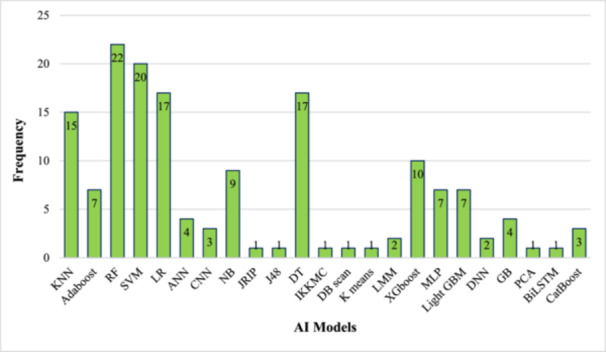
AI models for predicting ADHD in children and adolescents. AdaBoosting = Adaptive Boosting, AI = artificial intelligence, ANN = artificial neural network, BiLSTM = bidirectional long short‐term memory, CatBoost = categorical boosting, CNN = convolutional neural network, DBSCAN = density‐based spatial clustering of applications with noise, DNN = deep neural networks, DT = decision tree, GB = gradient boosting, KNN = K‐nearest neighbors, LMM = linear mixed models, LightGBM = light gradient boosting machine, LR = logistic regression, MLP = multilayer perceptron, NB = naive bayes, PCA = principal component analysis, RF = random forest, SVM = support vector machines, XGBoost= eXtreme gradient boosting.

Among the included studies, the most commonly used AI models for predicting ADHD in children and adolescents were Random Forest (RF) (*n* = 22; 14.1%) [30, 32, 34, 35, 38, 39, 41, 44, 46, 47, 52, 53, 54, 56, 57, 59, 60, 62, 63, 64, 65, 69], Support Vector Machine (SVM) (*n* = 20; 12.8%) [29, 30, 31, 32, 37, 44, 52, 53, 54, 55, 56, 57, 59, 60, 61, 62, 65, 67, 68, 69], Decision trees (DT) (*n* = 17; 10.9%) [29, 32, 37, 42, 44, 49, 52, 53, 54, 55, 56, 57, 58, 59, 60, 67, 69] and Logistic Regression (LR) (*n* = 17; 10.9%) [29, 30, 34, 38, 44, 51, 52, 53, 54, 55, 57, 59, 60, 61, 62, 65, 69] (Figure 3). Across all 42 studies, AI‐based methods were consistently shown to be effective for predicting ADHD in children and adolescents [28, 29, 30, 31, 32, 33, 34, 35, 36, 37, 38, 39, 40, 41, 42, 43, 44, 45, 46, 47, 48, 49, 50, 51, 52, 53, 54, 55, 56, 57, 58, 59, 60, 61, 62, 63, 64, 65, 66, 67, 68, 69]. In most studies (*n* = 27), multiple AI models were tested for comparison [28, 29, 30, 32, 34, 37, 38, 40, 41, 43, 44, 46, 52, 53, 54, 55, 56, 57, 59, 60, 61, 62, 63, 65, 67, 69]. Among these, RF (*n* = 9; 33.3%) [30, 41, 53, 56, 57, 59, 62, 63, 65] and LR (*n* = 5; 18.5%) [54, 55, 60, 61, 65] showed the highest predictive accuracy, averaging about 85%. Notably, a considerable number of recent studies (*n* = 9) employed deep learning architectures, including CNN [33, 43, 53], Artificial neural network (ANN) [36, 40, 52, 63], Bidirectional Long Short‐Term Memory (BiLSTM) [43], and Deep Neural Networks (DNN) [38, 66], achieving competitive or superior performance (mean of accuracy ≈ 89.90) compared to traditional machine learning models, particularly in studies utilizing neuroimaging or multimodal data sets.

**Figure 4:**
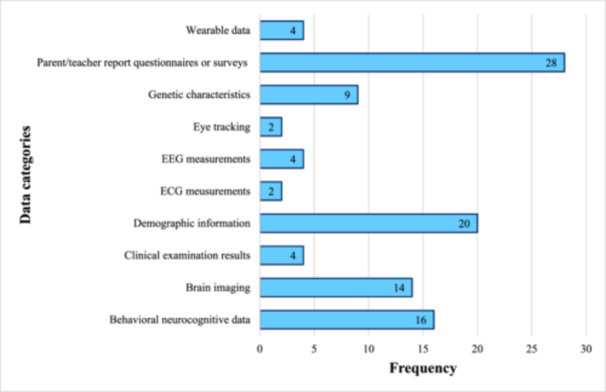
Types of data used in the included studies (*n* = 42). *Note:* The studies included in the figure utilized various data types to train AI models. These include wearable data (e.g., heart rate, physical activity); teacher and parent questionnaires (e.g., ADHD Rating Scale, Conners, Strengths and Difficulties Questionnaire); genetic features (e.g., polygenic risk scores, SNPs, rare CNVs); EEG, a non‐invasive measure of brain electrical activity associated with attention and impulse control; ECG, a non‐invasive measure of heart electrical activity to assess rate and rhythm; demographic information (age, sex, ethnicity, socioeconomic status, family background, IQ); clinical examination results (physical assessments and medical/laboratory tests); brain imaging (structural and functional scans, including MRI, fMRI, PET); and behavioral neurocognitive measures of attention, memory, executive function, and impulsivity.

In the included studies, the most commonly used data sources for ADHD prediction were parent‐ and teacher‐reported questionnaires and surveys (*n* = 28; 27.2%) [28, 31, 34, 35, 36, 39, 41, 42, 44, 46, 47, 48, 49, 50, 51, 52, 53, 54, 55, 56, 57, 59, 60, 61, 63, 65, 67, 68]. This was followed by demographic information such as age, sex, and IQ (*n* = 20; 19.4%) [29, 31, 33, 34, 35, 36, 38, 41, 42, 46, 48, 56, 57, 58, 59, 60, 61, 62, 67, 68], behavioral, cognitive, and neurological features of individuals with ADHD (*n* = 16; 15.5%) [29, 32, 33, 38, 40, 46, 48, 49, 50, 58, 60, 62, 64, 65, 66, 68], and neuroimaging data, including functional MRI (fMRI) (*n* = 14; 13.6%) [31, 33, 35, 36, 37, 42, 46, 48, 53, 57, 59, 60, 61, 66] (Figure 4).

Among the reviewed studies, 22 used closed‐source data sets (i.e., data collected directly from study participants or clinical settings), while 20 used open‐source data sets (i.e., publicly available databases) (Table 2). The number of features used for model development across studies ranged from 6 to 39,160. However, most studies (*n *= 24; 57.1%) used fewer than 99 features (Table 2).

**Table 2:** Number of features and types of data sets used in the included studies (*n* = 42).

Features		Studies, *n* (%)	References
Number of features	< 99	24 (57.1)	[28, 32, 34, 35, 38, 40, 41, 43, 45, 47, 48, 50, 51, 52, 53, 55, 56, 57, 59, 60, 63, 64, 67, 69]
	100–999	6 (14.3)	[29, 30, 42, 44, 62, 66]
	> 1000	6 (14.3)	[31, 33, 36, 37, 46, 65]
	Not reported	6 (14.3)	[39, 49, 54, 58, 61, 68]
Type of data set source	Closed	22 (52.4)	[28, 32, 34, 36, 38, 39, 40, 41, 42, 45, 47, 49, 50, 51, 53, 56, 58, 63, 66, 67, 68, 69]
	Opened	20 (47.6)	[29, 30, 31, 33, 35, 37, 43, 44, 46, 48, 52, 54, 55, 57, 59, 60, 61, 62, 64, 65]

The validation methods used in the included studies were mainly K‐fold cross‐validation (K‐fold CV) (*n* = 30; 71.4%) [28, 29, 31, 32, 33, 34, 38, 39, 41, 43, 44, 46, 47, 48, 51, 52, 53, 54, 55, 56, 57, 59, 60, 61, 62, 63, 65, 66, 67, 68]. Seven studies (16.7%) did not report their validation method [35, 36, 40, 49, 50, 58, 64]. Other validation methods included the Least Absolute Shrinkage and Selection Operator (LASSO) (*n* = 2; 4.8%) [30, 69], Leave‐one‐out cross‐validation (LOOCV) (*n *= 2; 4.8%) [37, 42], and leave‐one‐subject‐out cross‐validation (LOSO‐CV) (*n* = 1; 2.4%) [45]. The most frequently reported performance metrics were:

- Accuracy (*n* = 29; 25.4%), with values ranging from 67.00 to 99.60 and a mean of 87.74 ± 9.50;
- Sensitivity (recall) (*n* = 27; 23.7%), ranging from 50.00 to 99.70, with a mean of 87.11 ± 11.19;
- Specificity (*n* = 18; 15.8%), ranging from 65.00 to 99.70, with a mean of 84.97 ± 10.38.

These findings are summarized in Table 3.

**Table 3:** Frequency and mean values of performance evaluation metrics used in the included studies to assess the predictive ability of AI models for ADHD.

Performance measures	Definition	Frequency (%)	Mean ± SD (range)	References
Accuracy	Accuracy is the proportion of all predictions—both positive and negative—that the model classifies correctly. It reflects the overall correctness of the model across the entire data set.	29 (25.4)	87.74 ± 9.50 (67.0–99.60)	[28, 29, 31, 32, 33, 36, 38, 39, 41, 42, 43, 44, 45, 47, 49, 50, 51, 52, 53, 54, 55, 56, 57, 59, 61, 62, 63, 64, 67]
Sensitivity (recall)	Recall measures the proportion of actual positive cases that were correctly identified by the model, defined as the number of true positive predictions made by the model divided by the total number of actual positive cases in the data set.	27 (23.7)	87.11 ± 11.19 (50.0–99.70)	[28, 29, 32, 34, 36, 38, 39, 42, 43, 44, 45, 46, 47, 51, 52, 53, 54, 56, 57, 59, 61, 62, 63, 64, 65, 67, 68]
Specificity	Specificity in AI refers to the ability of a model to correctly identify negative cases, meaning it avoids falsely labeling healthy or non‐target individuals as positive. It reflects how well the system distinguishes true negatives from false positives.	18 (15.8)	84.97 ± 10.38 (65.0–99.70)	[32, 34, 38, 39, 42, 44, 45, 46, 47, 51, 53, 54, 56, 57, 61, 63, 64, 68]
AUC	AUC (Area Under the ROC Curve) measures how well an AI model separates positive from negative cases across all classification thresholds. It reflects the model's overall ability to discriminate between the two classes.	15 (13.2)	87.11 ± 9.24 (72.0–99.0)	[30, 31, 34, 36, 38, 46, 51, 52, 54, 56, 57, 59, 60, 64, 65]
F1‐score	F1‐score is used to balance precision and recall as a measure of a model's overall accuracy, defined as the harmonic mean of the model's precision and recall.	13 (11.4)	90.63 ± 8.56 (66.70–99.60)	[28, 29, 32, 34, 36, 44, 45, 47, 55, 59, 62, 65, 67]
Precision	Precision measures the proportion of positive predictions that were actually correct, defined as the number of true positive predictions made by the model divided by the total number of positive predictions made by the model.	12 (10.5)	92.59 ± 6.53 (79.0–100.0)	[28, 32, 36, 43, 44, 45, 47, 52, 59, 62, 65, 67]

### 3.3 Quality Assessment and Risk of Bias

The methodological quality of the included studies was assessed, and the results are summarized in Appendix C. All of the reviewed articles (*n* = 42) exhibited fair methodological quality with a low risk of bias [28, 29, 30, 31, 32, 33, 34, 35, 36, 37, 38, 39, 40, 41, 42, 43, 44, 45, 46, 47, 48, 49, 50, 51, 52, 53, 54, 55, 56, 57, 58, 59, 60, 61, 62, 63, 64, 65, 66, 67, 68, 69]. The average quality score across the studies was 83.03 ± 10.62 (Appendix C). Based on these results, all studies met the eligibility criteria and were included in this scoping review [28, 29, 30, 31, 32, 33, 34, 35, 36, 37, 38, 39, 40, 41, 42, 43, 44, 45, 46, 47, 48, 49, 50, 51, 52, 53, 54, 55, 56, 57, 58, 59, 60, 61, 62, 63, 64, 65, 66, 67, 68, 69].

## 4 Discussion

This study identified various AI models used to predict ADHD in children and adolescents. The findings show that most studies mainly used RF, SVM, LR, and DT models, with an average accuracy over 80% [29, 30, 31, 32, 34, 35, 37, 38, 39, 41, 42, 44, 46, 47, 49, 51, 52, 53, 54, 55, 56, 57, 58, 59, 60, 61, 62, 63, 64, 65, 67, 68, 69]. Several studies used multiple AI models at once for prediction [28, 29, 30, 32, 34, 37, 38, 40, 41, 43, 44, 46, 52, 53, 54, 55, 56, 57, 59, 60, 61, 62, 63, 65, 67, 69]. Among these, RF and LR had the highest accuracy. Supporting this, a recent review by Alhumaidi et al. identified RF as the most common method for predicting and managing various diseases, including cardiovascular, neurological, and cancerous conditions [70]. RF and other tree‐based ensemble models have emerged as particularly effective approaches for predicting ADHD in pediatric populations, owing to a confluence of methodological strengths that align closely with the inherent characteristics of clinical and behavioral data. Pediatric ADHD data sets are often heterogeneous and noisy—typically comprising multi‐informant reports (e.g., from parents, teachers, and clinicians)—and include mixed variable types, such as categorical (e.g., sex, ethnicity), ordinal (e.g., symptom severity ratings), and continuous features (e.g., age, cognitive test scores). RF models inherently accommodate this diversity without requiring extensive preprocessing, scaling, or encoding. Moreover, their ensemble architecture—built on bootstrap aggregation (bagging) and random feature subspace selection—confers robustness against overfitting, a critical advantage in studies with modest sample sizes (e.g., *n* = 103 in Shafna et al. [62]; *n* = 52 in Heller et al. [41]), where more complex models like deep neural networks often underperform due to limited data [71]. Importantly, RF also yields interpretable feature importance metrics, enabling clinicians to identify and prioritize key predictive factors (e.g., sleep disruption, inattentive behaviors at school), thereby facilitating transparent and collaborative clinical decision‐making [71]. These properties collectively account for the consistently high predictive performance and widespread adoption of RF across diverse early‐detection studies in this review [30, 41, 53, 56, 57, 59, 62, 63, 65], reinforcing its status as the most reliable and methodologically appropriate model for ADHD prediction in pediatric cohorts.

In contrast, a review of other studies shows that SVM and CNN are often reported as the most common approaches for managing ADHD [19, 20, 22, 72]. This difference in the choice of AI models between previous studies and current research may result from varying research goals. Specifically, earlier studies mainly focused on diagnosing ADHD, while the present study aimed to develop predictive models that can forecast the disorder in pediatric and adolescent populations. While deep learning models have shown promise in diagnostic neuroimaging tasks, their application in early prediction remains limited—primarily due to the lack of large‐scale, longitudinal, multimodal data sets in pre‐diagnostic populations. In contrast, traditional ML models excel with the structured, questionnaire‐based, and demographic data that dominate early‐risk research, offering a favorable balance of performance, interpretability, and practicality for real‐world screening. Indeed, among the 42 included studies, only nine employed deep learning [33, 36, 38, 40, 43, 52, 53, 63, 66]. These primarily used simple architectures (e.g., ANNs and CNNs) rather than complex models such as transformers or 3D‐CNNs, which are typically used in diagnostic fMRI studies. Among the studies using deep learning, Garcia‐Argibay et al. compared multiple models, including LR, RF, GB, XGBoost, NB, and DNN, and reported the DNN as the best‐performing model for predicting ADHD, achieving an accuracy of 69.0% [38]. Similarly, Lopez et al. evaluated LR, KNN, SVM, RF, XGBoost, ANN, and DT models, with the ANN showing the highest accuracy of 97.0% [52]. These findings indicate that deep learning models, though less commonly used in early‐prediction studies, can achieve competitive accuracy under certain conditions.

In the present study, most of the included studies used data from behavioral questionnaires and surveys [28, 31, 34, 35, 36, 39, 41, 42, 44, 46, 47, 48, 49, 50, 51, 52, 53, 54, 55, 56, 57, 59, 60, 61, 63, 65, 67, 68], demographic information [29, 31, 33, 34, 35, 36, 38, 41, 42, 46, 48, 56, 57, 58, 59, 60, 61, 62, 67, 68], and neurocognitive features [29, 32, 33, 38, 40, 46, 48, 49, 50, 58, 60, 62, 64, 65, 66, 68] to predict ADHD. These studies mainly focused on assessing behavioral symptoms and clinical signs in children, using standardized parent‐ and teacher‐reported tools like Conners' Rating Scales, 3rd edition (Conners‐3), and the ADHD Rating Scale (ADHD‐RS) to create data sets for developing predictive models of the disorder [34, 39, 41, 42, 47, 49, 50, 54, 56, 57, 60, 63, 67, 68]. While the majority of included studies relied on parent‐ and teacher‐reported questionnaires, these structured behavioral data can be effectively leveraged by AI models to detect complex, non‐linear patterns associated with ADHD risk that may not be apparent through conventional scoring. Similarly, studies using neuroimaging or cognitive features allow AI algorithms to extract subtle biomarkers and interrelationships across multiple brain regions or cognitive domains. Importantly, the application of AI to these data sets is not merely a replication of traditional assessment methods; rather, it enables early identification of children at risk before a formal clinical diagnosis, supports prioritization of follow‐up assessments, and provides a scalable, non‐invasive decision‐support tool for clinical and educational settings. From a clinical perspective, the reliance on questionnaire‐based data does not necessarily imply that AI merely replicates existing diagnostic routines. Traditional rating scales are typically interpreted using linear thresholds or clinician judgment, whereas AI models can extract multidimensional, non‐linear patterns that are imperceptible to human raters. These models can integrate multiple informants, demographic characteristics, developmental trajectories, and subtle behavioral signals to generate individualized risk probabilities rather than binary diagnostic decisions. Clinically, this enables earlier stratification of risk, prioritization of children who may otherwise be overlooked, and triage in high‐volume school or primary‐care environments where specialist resources are limited. The value of AI lies not in replacing clinical assessment but in enhancing scalability, reducing diagnostic delays, and enabling proactive monitoring in populations that have not yet entered the formal diagnostic pathway. As such, AI‐based prediction shifts the clinical paradigm from reactive diagnosis toward anticipatory early‐warning systems that support timely referral and intervention. The future integration of multimodal data, including behavioral, neurocognitive, neuroimaging, and genetic features, can further enhance predictive accuracy and clinical applicability. Conversely, earlier review studies have mainly used neuroimaging data, such as structural and fMRI [20], and electrophysiological signals, including EEG [22], for diagnosing ADHD. This difference in data types may stem from the different aims of prediction versus diagnosis. Specifically, early prediction of ADHD often requires easily accessible, non‐invasive, and scalable behavioral data, while a definitive clinical diagnosis tends to depend more on objective neurobiological evidence. Additionally, a substantial portion of the neuroimaging and electrophysiological data used in current research is derived from public repositories such as ADHD‐200 and the Adolescent Brain Cognitive Development (ABCD) study. Although these databases provide valuable standardized resources, they also present limitations related to sample size variability and differences in data acquisition protocols across sites, which can undermine reproducibility. The ADHD‐200 data set, in particular, has been widely adopted due to its open‐access nature, relatively large sample size, and suitability for benchmarking AI models. However, repeated reliance on this data set may result in overfitting to its specific characteristics, reduced generalizability to new populations, and the propagation of inherent biases. Dependence on a single data set further limits diversity in data sources and may impede progress toward integrating multimodal or longitudinal information that is essential for early ADHD prediction. Therefore, while ADHD‐200 remains a valuable foundational resource, future research should emphasize external validation and incorporate more diverse data sets to improve robustness and real‐world applicability. Furthermore, although multimodal data integration is often highlighted as essential for accurate ADHD prediction, our review found that fully multimodal approaches—integrating neuroimaging, behavioral, cognitive, and demographic data within a single predictive model—are still rare in early‐risk research. Of the 42 studies included, only eight [31, 33, 35, 42, 48, 57, 60, 61] incorporate all data types, including neuroimaging, behavioral, cognitive, and demographic data. This gap underscores a critical challenge: large‐scale, pre‐diagnostic cohorts (e.g., ABCD, National Survey of Children's Health (NSCH)) rarely include neuroimaging or genomic data, limiting the feasibility of complex multimodal modeling in real‐world screening contexts.

In the present study, most of the included publications focused on the 2023–2025 period, showing a significant and increasing scientific interest in using AI to predict ADHD [28, 29, 30, 32, 33, 34, 35, 36, 38, 39, 40, 43, 44, 45, 46, 47, 49, 51, 52, 55, 56, 57, 58, 59, 60, 62, 67, 69]. This rise in research activity may be due to the growing need for early diagnosis of the disorder and to major improvements in AI algorithms that enable more accurate analysis of complex behavioral data. In child and adolescent psychiatry, there is a continuous expansion in developing digital platforms that collect behavioral and demographic information in a comprehensive and ongoing way [73]. The increase in data from these platforms has created ideal conditions for applying advanced machine learning techniques, which can identify complex, non‐linear patterns across multiple data sources—patterns that traditional statistical methods often miss [74].

A substantial number of the studies included in this review used CV techniques, particularly K‐fold CV, to assess model performance [28, 29, 31, 32, 33, 34, 38, 39, 41, 43, 44, 46, 47, 48, 51, 52, 53, 54, 55, 56, 57, 59, 60, 61, 62, 63, 65, 66, 67, 68]. This finding aligns with earlier reviews in the field, which also relied on K‐fold CV as a common method for model validation [20, 22]. The frequent use of K‐fold cross‐validation among the studies can be explained by its ability to provide reliable, consistent, and nearly unbiased estimates of model performance, especially in research with limited sample sizes [75]. This approach ensures that each data sample is used exactly once in the test set and multiple times in the training sets across different folds, maximizing data usage and reducing the risk of overfitting. Consequently, K‐fold CV improves the robustness and generalizability of predictive models, making it a favored validation method in AI‐based clinical prediction studies.

Performance evaluation metrics such as accuracy, sensitivity (recall), and specificity were commonly used across most of the included studies to assess AI models for ADHD prediction [28, 29, 31, 32, 33, 34, 36, 38, 39, 41, 42, 43, 44, 45, 46, 47, 49, 50, 51, 52, 53, 54, 55, 56, 57, 59, 61, 62, 63, 64, 65, 67, 68]. The results of this study agree with previous research, which also primarily used accuracy, sensitivity, and specificity as standard metrics for evaluating AI models in diagnosing ADHD. This consistency is based on established methods for assessing ML models in clinical settings. These metrics are widely accepted in both clinical and AI research because they reliably measure a model's ability to correctly identify positive cases (individuals with ADHD) and negative cases (typically developing individuals) [76]. They offer clear insights into the balance between correct classifications and errors—especially false positives and false negatives—making them valuable for researchers developing and reporting ADHD prediction models. Additionally, the agreement among studies can partly be explained by similarities in data sources, preprocessing techniques, and target populations—particularly in studies that use standardized clinical assessments or neuroimaging data. This methodological consistency improves comparability and reproducibility across different research efforts. As a result, the regular application of these well‐known performance metrics is both methodologically appropriate and helpful for comparing, validating, and applying research findings across studies. Standardized reporting of accuracy, sensitivity, and specificity, therefore, plays an important role in advancing the practical use of AI models in pediatric mental health.

Overall, this study clearly shows a strong preference for machine learning algorithms that are relatively easy to interpret—such as RF, SVM, LR, and DT—in predicting ADHD in children and adolescents [29, 30, 31, 32, 34, 35, 37, 38, 39, 41, 42, 44, 46, 47, 49, 51, 52, 53, 54, 55, 56, 57, 58, 59, 60, 61, 62, 63, 64, 65, 67, 68, 69]. In addition to traditional machine learning models, some included studies [33, 36, 38, 40, 43, 52, 53, 63, 66] applied deep learning methods, such as CNNs, ANNs, BiLSTM, and DNNs. These models often demonstrated improved predictive performance, especially in studies using complex or multimodal data sets, suggesting that deep learning approaches are increasingly relevant and may complement traditional ML methods for early ADHD prediction. Also, incorporating multimodal data (such as neuroimaging, behavioral assessments, genetic markers, and demographic information) not only improves prediction but also increases the robustness and generalizability of AI models for ADHD risk assessment. For instance, Jaafar et al. [43] combined neuroimaging and behavioral data using deep learning architectures such as CNNs and hybrid models (CNN+BiLSTM), achieving higher accuracy than traditional ML models that rely on a single data source. Moreover, translating AI‐based ADHD prediction models into real‐world clinical practice requires careful consideration of several practical and regulatory factors. Beyond achieving high predictive accuracy, models must also offer sufficient interpretability for clinicians, integrate seamlessly into existing clinical workflows, and comply with the relevant ethical and regulatory standards in pediatric mental health. To facilitate clinical adoption, AI models should prioritize transparent feature importance and explainable outputs that allow clinicians to understand risk predictions. Integration with electronic health records and school‐based monitoring systems can streamline workflow, while adherence to regulatory guidelines and ethical standards—such as data privacy, bias mitigation, and validation across diverse populations—is essential. Pilot implementation studies and collaboration between AI developers, clinicians, and regulatory bodies are recommended to ensure safe, interpretable, and practical deployment of AI‐based ADHD risk prediction in real‐world pediatric settings. Although traditional machine‐learning methods such as RF, SVM, and LR provide more interpretable outputs, their clinical utility ultimately depends on how well their predictions align with clinician judgment and support patient management decisions. Building clinician trust, ensuring transparent reasoning, and designing systems that meaningfully fit within routine care processes remain essential steps. Addressing these challenges is critical to ensuring that advanced AI tools not only perform well in research settings but also deliver actionable value in early ADHD screening and intervention in real‐world pediatric environments.

Our findings also reveal that most studies mainly rely on standardized parent‐ and teacher‐completed questionnaires as the primary data source. While these tools are cost‐effective and easily accessible, they may be influenced by subjective reporting biases and often lack objective biological validation. However, the value of AI in this context lies not in replacing clinical judgment, but in enabling early, population‐level risk stratification using routinely collected data—long before formal diagnosis is considered. For instance, models trained on large cohort data (e.g., ABCD, NSCH, Early Development Instrument (EDI) [35, 36, 43, 46, 51, 53, 55, 59, 66] can identify at‐risk children in schools or primary care settings, facilitating timely referral and reducing diagnostic delays. Thus, even when using conventional inputs, AI shifts the paradigm from reactive diagnosis to proactive screening. The consistent methodological approaches across studies underscore the need to standardize data‐driven methods and to integrate multi‐dimensional data sources—such as neuroimaging, neuropsychological assessments, and genetic information—to enhance the validity, robustness, and broader applicability of predictive models. Importantly, AI‐driven prediction should be viewed as a decision‐support tool that complements, rather than replaces, clinical judgment. By providing actionable insights, such systems can help clinicians prioritize assessments and interventions for at‐risk children, potentially reducing diagnostic delays and supporting more timely and targeted clinical care.

The reviewed studies have several methodological limitations. Many models were at risk of overfitting, and dependence on publicly available data without controlling for potential biases in data collection. In addition, there was notable heterogeneity in how model performance was reported—ranging from accuracy‐only summaries to full ROC analyses. Only five of the 42 included studies reported 95% confidence intervals for their performance metrics, while the majority presented point estimates (e.g., accuracy values) without quantifying uncertainty. This lack of standardized reporting limits the ability to evaluate the reliability, comparability, and generalizability of the models. Despite these limitations, this scoping review provides a valuable framework for selecting algorithms and data sources in future research. It also emphasizes the need to develop more accurate, hybrid models based on multi‐modal evidence. These models have significant potential to enable early, non‐invasive, and reliable screening of ADHD in children and adolescents, and to improve outcomes through timely clinical and educational interventions.

### 4.1 Limitations and Future Directions

The present study has two key limitations that future research should address. First, only studies published in English were included, potentially excluding relevant research in other languages. Second, our search was confined to four major databases—PubMed, Scopus, Web of Science, and Embase—possibly leading to the omission of pertinent studies in other reputable sources. Future reviews can overcome these limitations by including non‐English publications and broadening their searches to more databases, thereby improving the comprehensiveness and generalizability of the findings. It is also important to clarify that our exclusion of prominent deep learning studies based on neuroimaging data was intentional and aligned with our scoping objective. These studies typically address diagnostic classification in already identified cases of ADHD versus controls. While methodologically valuable, they do not focus on prospective prediction in undiagnosed or community‐based pediatric populations—the core aim of this review. Thus, their omission reflects a deliberate scope boundary rather than a gap in literature coverage. Future work could bridge these two streams by integrating behavioral risk markers with neuroimaging biomarkers for hybrid early‐warning systems.

Beyond methodological considerations, several key challenges and opportunities remain for advancing AI‐based predictive models for ADHD. First, the integration of multi‐modal data—including neuroimaging, genetics, digital behavior, and clinical parameters—offers potential for more comprehensive and personalized predictions, though such data sets are currently scarce in pediatric populations. Second, hybrid approaches that combine interpretable machine learning with deep feature extraction, as well as advanced architectures like cross‐modal transformers and graph‐based fusion networks, may enhance both predictive accuracy and clinical utility. Third, standardizing evaluation metrics, data sets, and feature selection methods is critical to improve comparability and reproducibility across studies. Finally, pragmatic early‐warning systems leveraging accessible behavioral and demographic data can provide immediate clinical value, while multimodal integration may be reserved for high‐risk subgroups. Addressing these challenges will support the development of more robust, scalable, and clinically applicable tools for early, non‐invasive ADHD prediction.

## 5 Conclusion

This scoping review investigated AI models and algorithms used to predict, detect early, and classify ADHD in children and adolescents. The results demonstrate that AI—particularly through machine learning algorithms such as RF, SVM, LR, and DT—has significant potential to enhance prediction accuracy and support clinical decisions. These models have consistently demonstrated acceptable levels of accuracy, sensitivity, and specificity in early ADHD detection, which could help reduce diagnostic delays, enable faster interventions, and ultimately lower the clinical and societal impact of the disorder. Additionally, using standardized parent‐ and teacher‐reported questionnaires as the main data sources makes these models cost‐effective and scalable for use in schools and primary healthcare settings. Nevertheless, several issues remain, including variations across study populations, inconsistent input features, limited objective biomarkers, and a lack of transparency and open access, which may limit their generalizability and practical utility. Future research should aim to enhance model performance by integrating multimodal data, such as neuroimaging, behavioral markers, and genetic information, while also validating models across diverse populations, standardizing data collection methods, and developing reliable and interpretable AI systems that can be integrated into child and adolescent mental health settings. Ultimately, AI should serve as a transparent, workflow‐integrated, and ethically governed supportive tool for mental health professionals—not replace them—ensuring that clinical expertise, family context, and child development remain at the center of care.

## Author Contributions

A.A., F.S., H.S., and M.G.h. were responsible for designing the study, analyzing the data, interpreting the results, and writing the manuscript. All authors have read and approved the final version of the manuscript. H.S. had full access to all of the data in this study and takes complete responsibility for the integrity of the data and the accuracy of the data analysis.

## Ethics Statement

The study was approved by the Research Ethics Committee of Kerman University of Medical Sciences (Code of Ethics: IR.KMU.REC.1403.509).

## Conflicts of Interest

The authors declare no conflicts of interest.

## Transparency Statement

The corresponding authors, Hamidreza Samzadeh and Mohammad Mehdi Ghaemi, affirm that this manuscript is an honest, accurate, and transparent account of the study being reported; that no important aspects of the study have been omitted; and that any discrepancies from the study as planned (and, if relevant, registered) have been explained.

## Supporting information

Appendix A.docx.

Appendix B.docx.

Appendix C.docx.

## Data Availability

All data generated or analyzed during this study are included in this published article. Additional information about the data sets used and/or analyzed during this study is available from the corresponding author upon reasonable request.
