# Supplementary material for: Predicting ADHD in Children and Adolescents With Artificial Intelligence: A Scoping Review of Common Models
Source: Health Sci Rep. 2025 Dec 21;8(12):e71679. doi: 10.1002/hsr2.71679 (PMC12719396; doi:10.1002/hsr2.71679)
Supplement: Supplementary file 1 — Appendix A.docx. [file HSR2-8-e71679-s003.docx]

**Appendix A.** Search strategy

**Group A:** “Artificial intelligence” OR “machine learning” OR “deep learning” OR “AI Technologies” OR “data mining”

**Group B:** ADHD OR ADDH OR “attention deficit disorders with Hyperactivity” OR “attention deficit hyperactivity disorders” OR “attention deficit hyperactivity disorder”

**Final search Strategy:** Group A AND Group B
