# Supplementary material for: Predicting ADHD in Children and Adolescents With Artificial Intelligence: A Scoping Review of Common Models
Source: Health Sci Rep. 2025 Dec 21;8(12):e71679. doi: 10.1002/hsr2.71679 (PMC12719396; doi:10.1002/hsr2.71679)
Supplement: Supplementary file 2 — Appendix B.docx. [file HSR2-8-e71679-s002.docx]

**Appendix B.** Characteristics of included studies (n=42)

| **No** | **Author (Year)** | **Country** | **Data set** | **AI Models** | **Performance evaluation measures** | **Findings** |
| --- | --- | --- | --- | --- | --- | --- |
| 1 | Ali et al., (2024) [28] | Iraq | Data from surveying parents (153 children aged 6–12 years) | KNN, AdaBoost | Accuracy, recall, F1-score, precision | The best-fitting model was the KNN.  Accuracy= 96.5% for unbalanced data  Accuracy= 98.59% for balanced data |
| 2 | Archana et al., (2025) [29] | India | Data from NSCH (42778 children aged 0-17 years) | DT, NB, LR, KNN, SVM | Accuracy, sensitivity, F1-score | The best-fitting model was the DT.  Accuracy= 90.13%, sensitivity= 89.47%, F1-score= 94.33% |
| 3 | Barnett et al., (2025) [30] | USA | Data from PGC cohort studies (2455 ADHD cases and 8432 controls with a mean age of>13 years) | RF, SVM, KNN, LR | AUC, Lasso | The final best-performing model was the RF.  AUC= 0.72 (95% CI: 0.70–0.74) |
| 4 | Bohland et al., (2012) [31] | USA | ADHD-200 Dataset (362 children and adolescents diagnosed with ADHD and 585 typically developing controls aged 7-21 years) | Linear SVM | ROC, AUC, accuracy | AUC ≈ 0.72-0.81 |
| 5 | Cha et al., (2025) [32] | Republic of Korea | Data from 15 clinics in South Korea (11429 children with a mean age of 11-14 years) | NB, KNN, DT, SVM, RF, CatBoost, LightGBM, GB | Accuracy, sensitivity, specificity, F1-score, precision, recall | The best-fitting model was the GB.  Accuracy= 98.0%, sensitivity= 98.0%, specificity= 96.0%, F1-score= 98.0% |
| 6 | Chen et al., (2023) [33] | China | Data from images of faces, eyes, and gaze location (258 children with a mean age ≈ of 9 years) | CNN | Accuracy, adjusted accuracy | Accuracy= 44.98%, accuracy (adjusted)= 90.02% |
| 7 | Choi et al., (2025) [34] | Republic of Korea | Data from two tertiary hospitals in South Korea (323 with ADHD, 323 typically developing controls with a mean age ≈ of 9.5 years) | RF, XGBoost, LR | AUROC, sensitivity, specificity, F1-score | The best-fitting model was the XGBoost.  (median 87%, IQR 85.2–88.5%)  AUROC= 0.969 (95% CI 96.2–97.7%), sensitivity= 91.6% (95% CI 89.7–93.7%), specificity= 92.0% (95% CI 90.1–93.9%), F1-score= 91.7% (95% CI 89.8–93.6%. |
| 8 | Choi et al., (2025) [35] | Republic of Korea | Data from the ABCD cohort study (11880 children aged 9-10 years) | RF | Heterogeneity Index, Average Treatment Effect (ATE) | ATE= 1.29, heterogeneity index= 1.26- 1.35 |
| 9 | De Lacy et al., )2023) [36] | USA | Data from the ABCD cohort study (11880 children aged 9-10 years) | ANN | Accuracy, precision, recall, AUC, ROC | Accuracy=93.7%, precision=91.8%, recall=91.6%, AUROC=0.985 |
| 10 | Garcia et al., (2017) [37] | Brazil | Data from three datasets aged between 7 and 21 years old: KKI (58 control and 20 ADHD samples), NeuroIMAGE (22 control and 17 ADHD samples), NYU (91 control and 96 ADHD samples), and Peking (93 control and 57 ADHD samples) | SVM, KNN, MLP, C4.5, NB | PCC, Wilcoxon, LASSO, RFE, MRMD | The best-fitting model was the C4.5.  KKI (PCC=0.65, Wilcoxon= 0.68, LASSO= 0.47, RFE= 0.65, MRMD= 0.47)  NeuralIMAGE (PCC=0.81, Wilcoxon= 0.66, LASSO= 0.53, RFE= 0.69, MRMD= 0.87)  NYU (PCC=0.58, Wilcoxon= 0.52, LASSO= 0.57, RFE= 0.52, MRMD= 0.57) |
| 11 | Garcia-Argibay et al., (2023) [38] | Sweden | Data from born and living in Sweden (238696 children aged 14-18 years) | LR, RF, GB, XGboost, NB, DNN | Accuracy, sensitivity, specificity, AUC | The best-fitting model was the DNN.  AUC= 0.75, 95% CI (0.74–0.76), accuracy= 69.0%, sensitivity= 71.66%, specificity= 65.0% |
| 12 | Goh et al., (2023) [39] | USA | Data from the Oregon ADHD-1000 Cohort (399 children aged 7-19 years) | RF | Accuracy, sensitivity, specificity, PPV, NPV | In baseline: accuracy: 92.0%, sensitivity= 97.0%, specificity= 86.0%, PPV= 90.0%, NPV= 96.0%  Five years later: accuracy: 93.0%, sensitivity= 89.0%, specificity= 95.0%, PPV= 92.0%, NPV= 93.0% |
| 13 | Guigou et al., (2025) [40] | France | Data from the EPIDIA4kids study (24 children aged 7–12 years) | LMM, PCA, ANN | P-values, effect sizes | The best-fitting model was the PCA.  working memory (p <0.0001), processing speed (p<0.0001), visuospatial skills (p=0.003), inattention (p=0.04), achievement (p = 0.04) |
| 14 | Heller et al., (2013) [41] | USA | Two outpatient clinics (52 children and adolescents aged 6 -17 years) | AdaBoost, JRip, J48, RF | Accuracy | The best-fitting model was the RF. Accuracy= 75.0-78.0% |
| 15 | Itani et al., (2019) [42] | Belgium | ADHD-200 Dataset (362 children and adolescents diagnosed with ADHD and 585 typically developing controls aged 7-21 years) | DT | Accuracy, specificity, sensitivity | Accuracy= 73.2% |
| 16 | Jaafar et al., (2025) [43] | Iraq | EEG dataset from IEEE DataPort (children aged 5–16 years) | CNN, BiLSTM, Hybrid Model (CNN+BiLSTM) | Accuracy, recall, precision | The best-fitting model was the CNN+BiLSTM hybrid model.  Accuracy= 98.69%, recall= 96.88%, precision= 95.55% |
| 17 | Khandelwal et al., (2025) [44] | India | Data from the Child Health Data dataset (59963 children aged 3–17 years) | KNN, DT, NB, MLP, SVM, LR, RF, GB | Accuracy, precision, recall, specificity, F1-score | The best-fitting model was the DT.  Accuracy= 99.6%, recall= 99.7%, precision= 99.4%, specificity= 99.7%, F1-score= 99.6% |
| 18 | Kim et al., (2025) [45] | Republic of Korea | Data from Daegu Catholic University, Seoul National University Hospital, and Hanyang University Hospital (168 children aged 8–15 years) | XGBoost | Accuracy, F1-score, precision, recall, specificity | Accuracy= 90.81%, F1-score= 93.47%, precision= 92.58%, recall= 94.37%, specificity= 82.64% |
| 19 | Kim at al., )2023) [46] | Republic of Korea | Data from the ABCD cohort study (11880 children aged 9-10 years) | RF, XGBoost, LightGBM | AUC, sensitivity, specificity, PPV, NPV | The best-fitting model was the light GBM  AUC= 0.791 (95% CI, 0.790-0.792), sensitivity= 71.8% (95% CI, 0.715-0.722), specificity= 71.6% (95% CI, 0.715-0.717), PPV= 15.2% (95% CI, 0.152-0.153), NPV= 97.3% (95% CI, 0.972-0.973) |
| 20 | Komijani et al., (2025) [47] | USA | Data from the Mapping Impulsivity’s Neurodevelopmental Trajectory study (272 adolescents and young adults aged 12-30 years) | RF | Accuracy, F1-score, precision, recall, specificity | Accuracy= 86.0%, precision= 100%, recall= 50.0%, F1-score= 66.7.0% |
| 21 | Lalithambigai et al., (2019) [48] | India | ADHD-200 Dataset (362 children and adolescents diagnosed with ADHD and 585 typically developing controls aged 7-21 years) | IKKMC, K-Means DBSCAN | Accuracy | The best fitting model was the IKKMC, Accuracy= 88.0% |
| 22 | Lavigne et al., (2024) [49] | USA | Data from preschool children (796 children with a mean age of 4.44 years) | DT | Accuracy | Accuracy=70.60% |
| 23 | Leikauf, et al., )2017) [50] | Australia | Data from cognitive testing and clinical rating scales (198 children aged 6-17 years) | LMM | Accuracy | Accuracy= 89.0% |
| 24 | Liu et al., (2024) [51] | Canada | Data from EDI (69486 children aged 5-6 years) | LR | AUC, accuracy, sensitivity, specificity | Accuracy= 74.5%, AUC= 0.811, sensitivity= 71.7%, specificity= 77.3% |
| 25 | Lopez et al., (2024) [52] | USA | Data from CAP’s dataset (146 medication-naive children with ADHD and 209 controls with a mean age of 7.3 years) | LR, KNN, SVM, RF, XGBoost, ANN, DT | Accuracy, precision, recall, AUC, ROC | The best-fitting model was the ANN.  Accuracy= 97.0%, AUC= 0.99, recall= 97.0%, precision= 98.0% |
| 26 | Maniruzzaman et al. (2022) [53] | Japan | Data from NSCH, 2018–2019 (45779 children aged 3–17 years) | LR, RF, NB, DT, KNN, MLP, SVM, 1D CNN | Accuracy, sensitivity, specificity, PPV, NPV, ROC | The best-fitting model was the RF. Accuracy=85.5%, sensitivity=84.4%, specificity=86.4%, AUC=0.94. |
| 27 | Mooney et al., (2021) [54] | USA | Data from the Oregon-ADHD-1000 case-control cohort (1423 children aged 7-11 years) | LR, DT, RF, SVM, GBDT | Accuracy, sensitivity, AUC-ROC, PPV, specificity | The best-fitting model was the LR.  Accuracy= 87.4%, AUC-ROC= 0.942, PPV= 84.3%, specificity= 82.9%, sensitivity= 91.7% |
| 28 | Namasse et al., (2025) [55] | North Africa | Data from the NSCH 2022 dataset (39924 children aged 3–17 years) | LR, DT, KNN, SVM, MLP | Accuracy, F1-score | The best-fitting model was the LR.  Accuracy= 99.0%, F1-score= 99.6% |
| 29 | Navarro-Soria et al., (2025) [56] | Spain | Data from child and adolescent mental health units in the province of Alicante (406 children aged 6–16 years) | DT, RF, AdaBoost, XGBoost, CatBoost, LightGBM, SVM, MLP, NB, KNN | Accuracy, AUC, sensitivity, specificity | The best-fitting model was the RF.  Accuracy= 90.0%, AUC= 0.94, sensitivity= 91.0%, specificity= 92.0% |
| 30 | Qin et al., (2025) [57] | China | ADHD-200 Dataset (362 children and adolescents diagnosed with ADHD and 585 typically developing controls aged 7-21 years) | RF, SVM, LR, AdaBoost, DT, XGBoost, KNN | Accuracy, AUC, sensitivity, specificity | The best-fitting model was the RF.  Accuracy = 95.0%, sensitivity= 95.0%, specificity= 95.0%, F1-score= 95.0%, AUC= 0.99. |
| 31 | Quintero-López et al., (2023) [58] | Columbia | Data from 712 schoolchildren (564 boys and 148 girls aged 6-13 years) | DT | Not mentioned | Probability of predicting ADHD 29.63% |
| 32 | Rahman, (2025) [59] | USA | Data from the ABCD cohort study (11880 children aged 9-10 years) | DT, RF, NB, AdaBoost, Light GBM, LR, SVM, KNN | Accuracy, precision, recall, F1-score, AUC | The best-fitting model was the RF.  Accuracy= 89.24%, precision= 86.73%, recall= 88.51%, F1-score= 87.61%, AUC= 0.95 |
| 33 | Santarrosa-López et al., (2025) [60] | Mexico | Hyperaktiv dataset (n=103) + ADHD-200 Dataset (n=776) + EEG Data for ADHD (n=121) | LR, CatBoost, SVM, GB, AdaBoost, RF, DT, LightGBM, XGBoost, KNN | Accuracy, precision, recall, F1-score, ROC-AUC | The best-fitting model was the LR.  Accuracy= 90.47%, precision= 90.90%, recall= 91.66%, F1-score= 90.00%, ROC-AUC= 0.9166 |
| 34 | Sato et al., )2012) [61] | Brazil | ADHD-200 Dataset (362 children and adolescents diagnosed with ADHD and 585 typically developing controls aged 7-21 years) | Linear SVM, LR | Sensitivity, specificity, accuracy | The best-fitting model was the LR.  Accuracy= 67.0% |
| 35 | Shafna et al., (2024) [62] | India | Hyperaktiv dataset (51 patients with ADHD and 52 clinical controls with a mean age 10.2 years) | LR, RF, XGBoost, LightGBM, SVM | Accuracy, precision, F1-score, recall | The best-fitting model was the RF.  Accuracy= 84.0%, precision= 79.0%, F1-score= 85.0%, recall= 92.0%. |
| 36 | Slobodin et al., )2020) [63] | Israel | Data from out-patient pediatric clinics of a Neuro-Cognitive Centre (458 children aged 6–12 years) | RF, ANN | Accuracy, sensitivity, specificity | The best-fitting model was the RF.  Accuracy= 87% (%95 CI = 81 to 93%), sensitivity= 89% (%95 CI = 83 to 95%), specificity= 84% (%95 CI = 76 to 92%) |
| 37 | Sudre et al., (2021) [64] | USA | Data from a cohort study (362 children with a mean age of 4.8 years) | RF | AUC, sensitivity, specificity | AUC= 0.83, sensitivity= 75.0%, specificity=67.0% |
| 38 | Ter-Minassian et al., (2021) [65] | UK | Data from educational services (57149 children aged 4-7 years) | LR, RF, SVM, NB, MLP | AUC, precision, recall, F1-score | The best-fitting models were the RF and LR.  RF: AUC= 0.86, precision =98.0%, recall= 81.0%, F1-score= 88.0%  LR: AUC= 0.88, precision= 98.0%, recall= 81.0%, F1-score=88.0% |
| 39 | Uyulan et al., (2022) [66] | Turkey | Data from Bursa Yuksek Ihtisas Research and Training Hospital (433 children and adolescents with a mean age of 6 years) | DNN | R-value, MSE, MAE | R-value=91%, MSE=1.0864*10, MAE=0.0068 |
| 40 | Wang et al., (2024) [67] | China | Data from the outpatient clinic (202 children aged 3-14 years) | DT, KNN, SVM | Accuracy, precision, recall, F1-score | The best-fitting model was the SVM.  Accuracy= 93.1%, precision =86.6%, recall= 92.1%%, F1-score= 89.2% |
| 41 | Yasumura et al., (2020) [68] | Japan | Data from six facilities in Japan (216 children with a mean age≈ 10 years) | SVM | Sensitivity, specificity | Sensitivity= 87.1%, specificity= 83.78% |
| 42 | Zhang et al., (2025) [69] | China | Data from a cohort study (740 children aged 1 to 18 years) | AdaBoost, LR, RF, GBM, XGBoost, SVM, KNN, MLP, DT | ROC, PRC, Lasso | The best-fitting model was the GBM.  ROC= 0.91, PRC= 0.95 |

**Note:** The statistical values reported in this appendix (including any P values or significance indicators) are extracted directly from the original primary studies. No hypothesis testing was conducted by the authors of this scoping review, and no P values were generated or recalculated. The reporting format reflects the original studies’ conventions.

**Abbreviations:** AdaBoosting= Adaptive Boosting; KNN= K-Nearest Neighbors ; DT= Decision Tree; NB= Naive Bayes; SVM= Support Vector Machines; ABCD= Adolescent Brain Cognitive Development; NSCH= National Survey of Children’s Health ; PGC= Psychiatric Genomics Consortium ; ADHD= Attention Deficit Hyperactivity Disorder; CPT= Continuous Performance Test; RF= Random Forest; LR= Logistic Regression; CatBoost= Categorical Boosting; LightGBM= Light Gradient Boosting Machine; GB= Gradient Boosting; CNN= Convolutional Neural Network; XGBoost= eXtreme Gradient Boosting; ANN= Artificial Neural Network; AUC= Area Under the Curve ; ROC= Receiver Operating Characteristic; MLP= Multilayer Perceptron; DBSCAN= Density-based spatial clustering of applications with noise; PPV= Positive Predictive Values; NPV= Negative Predictive Values; LMM= Linear Mixed Models; PCA= Principal Component Analysis; EDI= Early Development Instrument; BiLSTM= Bidirectional Long Short-Term Memory; GBDT= Gradient-Boosted Decision Trees; DNN= Deep Neural Networks; MSE= Mean Squared Error ; MAE= Mean Absolute Error;
