# Supplementary material for: Predicting ADHD in Children and Adolescents With Artificial Intelligence: A Scoping Review of Common Models
Source: Health Sci Rep. 2025 Dec 21;8(12):e71679. doi: 10.1002/hsr2.71679 (PMC12719396; doi:10.1002/hsr2.71679)
Supplement: Supplementary file 3 — Appendix C.docx. [file HSR2-8-e71679-s001.docx]

**Appendix C: Quality assessments using Joanna Briggs Institute (JBI) appraisal checklist**

**Abbreviations:** 1 = Yes; 0 = No; U = Unclear; NA = Not Applicable

**Criteria used to rank the risk of bias**

I) ≤49%=High risk of Bias

II) 50% and 69%=Moderate risk of Bias

III) Above 70%=Low risk of Bias

| **No** | **Author (Year)** | **Items on Joanna Briggs Institute** | | | | | | | | **Raw score and %** | **Risk** |
| --- | --- | --- | --- | --- | --- | --- | --- | --- | --- | --- | --- |
|  |  | **Q1** | **Q2** | **Q3** | **Q4** | **Q5** | **Q6** | **Q7** | **Q8** |  |  |
| 1 | Ali et al., (2024) [28] | 1 | 1 | 1 | 1 | 0 | 0 | 1 | 1 | 6/8=75% | Low |
| 2 | Archana et al., (2025) [29] | 1 | 1 | 1 | 1 | 0 | 0 | 1 | 1 | 6/8=75% | Low |
| 3 | Barnett et al., (2025) [30] | 1 | 1 | 1 | 1 | 1 | 1 | 1 | 1 | 8/8=100% | Low |
| 4 | Bohland et al., (2012) [31] | 1 | 1 | 1 | 1 | 1 | 1 | 1 | 1 | 8/8=100% | Low |
| 5 | Cha et al., (2025) [32] | 1 | 1 | 1 | 1 | 0 | 0 | 1 | 1 | 6/8=75% | Low |
| 6 | Chen et al., (2023) [33] | 1 | 1 | 1 | 1 | 1 | 1 | 1 | 1 | 8/8=100% | Low |
| 7 | Choi et al., (2025) [34] | 1 | 1 | 1 | 1 | 1 | 1 | 1 | 1 | 8/8=100% | Low |
| 8 | Choi et al., (2025) [35] | 1 | 1 | 1 | 1 | 1 | 1 | 1 | 1 | 8/8=100% | Low |
| 9 | De Lacy et al., )2023) [36] | 1 | 1 | 1 | 1 | 0 | 0 | 1 | 1 | 6/8=75% | Low |
| 10 | Garcia et al., (2017) [37] | 1 | 1 | 1 | 1 | 1 | 0 | 1 | 1 | 7/8=87/5% | Low |
| 11 | Garcia-Argibay et al., (2023) [38] | 1 | 1 | 1 | 1 | 0 | 0 | 1 | 1 | 6/8=75% | Low |
| 12 | Goh et al., (2023) [39] | 1 | 1 | 1 | 1 | 1 | 1 | 1 | 1 | 8/8=100% | Low |
| 13 | Guigou et al., (2025) [40] | 1 | 1 | 1 | 1 | 1 | 0 | 1 | 1 | 7/8=87.5% | Low |
| 14 | Heller et al., (2013) [41] | 1 | 1 | 1 | 1 | 0 | 0 | 1 | 1 | 6/8=75% | Low |
| 15 | Itani et al., (2019) [42] | 1 | 1 | 1 | 1 | 0 | 0 | 1 | 1 | 6/8=75% | Low |
| 16 | Jaafar et al., (2025) [43] | 1 | 1 | 1 | 1 | 0 | 0 | 1 | 1 | 6/8=75% | Low |
| 17 | Khandelwal et al., (2025) [44] | 1 | 1 | 1 | 1 | 0 | 0 | 1 | 1 | 6/8=75% | Low |
| 18 | Kim et al., (2025) [45] | 1 | 1 | 1 | 1 | 0 | 0 | 1 | 1 | 6/8=75% | Low |
| 19 | Kim at al., )2023) [46] | 1 | 1 | 1 | 1 | 1 | 0 | 1 | 1 | 7/8=87.5% | Low |
| 20 | Komijani et al., (2025) [47] | 1 | 1 | 1 | 1 | 0 | 0 | 1 | 1 | 6/8=75% | Low |
| 21 | Lalithambigai et al., (2019) [48] | 1 | 1 | 1 | 1 | 0 | 0 | 1 | 1 | 6/8=75% | Low |
| 22 | Lavigne et al., (2024) [49] | 1 | 1 | 1 | 1 | 1 | 0 | 1 | 1 | 7/8=87.5% | Low |
| 23 | Leikauf, et al., )2017) [50] | 1 | 1 | 1 | 1 | 1 | 1 | 1 | 1 | 8/8=100% | Low |
| 24 | Liu et al., (2024) [51] | 1 | 1 | 1 | 1 | 1 | 0 | 1 | 1 | 7/8=87.5% | Low |
| 25 | Lopez et al., (2024) [52] | 1 | 1 | 1 | 1 | 1 | 1 | 1 | 1 | 8/8=100% | Low |
| 26 | Maniruzzaman et al., (2022) [53] | 1 | 1 | 1 | 1 | 1 | 0 | 1 | 1 | 7/8=87.5% | Low |
| 27 | Mooney et al., (2021) [54] | 1 | 1 | 1 | 1 | 1 | 1 | 1 | 1 | 8/8=100% | Low |
| 28 | Namasse et al., (2025) [55] | 1 | 1 | 1 | 1 | 0 | 0 | 1 | 1 | 6/8=75% | Low |
| 29 | Navarro-Soria et al., (2025) [56] | 1 | 1 | 1 | 1 | 0 | 0 | 1 | 1 | 6/8=75% | Low |
| 30 | Qin et al., (2025) [57] | 1 | 1 | 1 | 1 | 0 | 0 | 1 | 1 | 6/8=75% | Low |
| 31 | Quintero-López et al., (2023) [58] | 1 | 1 | 1 | 1 | 0 | 0 | 1 | 1 | 6/8=75% | Low |
| 32 | Rahman, (2025) [59] | 1 | 1 | 1 | 1 | 0 | 0 | 1 | 1 | 6/8=75% | Low |
| 33 | Santarrosa-López et al., (2025) [60] | 1 | 1 | 1 | 1 | 0 | 0 | 1 | 1 | 6/8=75% | Low |
| 34 | Sato et al., )2012) [61] | 1 | 1 | 1 | 1 | 0 | 0 | 1 | 1 | 6/8=75% | Low |
| 35 | Shafna et al., (2024) [62] | 1 | 1 | 1 | 1 | 0 | 0 | 1 | 1 | 6/8=75% | Low |
| 36 | Slobodin et al., )2020) [63] | 1 | 1 | 1 | 1 | 0 | 0 | 1 | 1 | 6/8=75% | Low |
| 37 | Sudre et al., (2021) [64] | 1 | 1 | 1 | 1 | 1 | 0 | 1 | 1 | 7/8=87.5% | Low |
| 38 | Ter-Minassian et al., (2021) [65] | 1 | 1 | 1 | 1 | 1 | 1 | 1 | 1 | 8/8=100% | Low |
| 39 | Uyulan et al., (2022) [66] | 1 | 1 | 1 | 1 | 0 | 0 | 1 | 1 | 6/8=75% | Low |
| 40 | Wang et al., (2024) [67] | 1 | 1 | 1 | 1 | 0 | 0 | 1 | 1 | 6/8=75% | Low |
| 41 | Yasumura et al., (2020) [68] | 1 | 1 | 1 | 1 | 0 | 0 | 1 | 1 | 6/8=75% | Low |
| 42 | Zhang et al., (2025) [69] | 1 | 1 | 1 | 1 | 0 | 0 | 1 | 1 | 6/8=75% | Low |

**Questions for cross-sectional studies:** Q1: Were the criteria for inclusion in the sample clearly defined?; Q2: Were the study subjects and the setting described in detail?; Q3: Was the exposure measured in a valid and reliable way?; Q4: Were objective, standard criteria used for measurement of the condition?; Q5: Were confounding factors identified?; Q6: Were strategies to deal with confounding factors stated?; Q7: Were the outcomes measured in a valid and reliable way?; Q8: Was appropriate statistical analysis used?
